# The Management of Retinal Detachment: Techniques and Perspectives

**DOI:** 10.1155/2017/5807653

**Published:** 2017-10-23

**Authors:** Elad Moisseiev, Anat Loewenstein, Ala Moshiri, Glenn Yiu

**Affiliations:** ^1^Department of Ophthalmology, Tel Aviv Medical Center, Affiliated to the Sackler School of Medicine, Tel Aviv University, Tel Aviv, Israel; ^2^Department of Ophthalmology & Vision Science, University of California Davis Eye Center, Sacramento, CA, USA

Retinal detachments are frequently encountered by ophthalmologists of all subspecialties and are managed by all vitreoretinal surgeons. Over the past century, the treatment of retinal detachment has been revolutionized, and it has evolved from an incurable untreatable condition leading to irreversible vision loss to a repairable occurrence after which visual recovery is possible. As technology and repair techniques continued to evolve, a myriad of therapeutic options are now available for the management of retinal detachments. Although retinal detachments are common, there is still some controversy on the optimal treatment options for different types and presentations of retinal detachment.

This special issue was intended to serve as a platform for sharing current data and new innovations in the management of retinal detachments. Twelve manuscripts have been selected for inclusion in this special issue, authored by ophthalmologists from all over the world, and they encompass the newest developments and innovations in the treatment of retinal detachment.

Improving the outcomes of retinal detachment repair surgery was a common theme of the manuscripts submitted to this special issue. S. Deuchler et al. investigated the risk for retinal redetachment in difficult cases with proliferative vitreoretinopathy or tractional retinal detachment that required silicone oil tamponade. From their detailed analysis of all factors related to these surgeries, they have found that with current techniques, the rate of retinal redetachment may be as low as 7% and suggest that following a standard operating procedure may help achieve the best results. Additionally, they found that better outcomes were achieved by more experienced surgeons and suggest that additional simulator training may improve the outcomes of more novice surgeons. K. Y. Pak et al. report a large comparative study, in which pars plana vitrectomy with gas tamponade for the repair of retinal detachment was compared to the surgery using only air for tamponade. In cases with air tamponade, visual recovery was faster and recurrent detachments were identified sooner, with no difference in the overall success rates. Based on these results, it seems that in eyes with uncomplicated retinal detachment, air tamponade may be considered. Another comparative study by S. Rizzo et al. compared 27-gauge and 25-gauge vitrectomy for the treatment of primary rhegmatogenous retinal detachments. No differences in the outcomes or surgical times were noted between the two, indicating that 27-gauge instrumentation is safe and effective for retinal detachment surgery. As this instrumentation is currently at the forefront of vitreoretinal surgery, it is likely that more surgeons will transition to it in the future and even more complicated cases can be performed using it. Z. Lin et al. reported a very large series with over 500 cases of rhegmatogenous retinal detachment repair surgeries, focusing on the safety and efficacy of adjustable postoperative position. Patients were instructed to maintain postoperative positioning based on the location of the breaks: those with superior and lateral break location were allowed to have facedown position or lateral decubitus position postoperatively and those with inferior break location were allowed to have facedown position. The reattachment and visual recovery rates were excellent, indicating that tailoring the postoperative position appropriately according to retinal break locations may be advantageous. R. Rejdak et al. investigated the outcomes of retinal detachment repair in children, which are frequently very challenging. They report that most cases also required lensectomy and intraocular lens implantation, as well as silicone oil tamponade. Retinal reattachment was achieved in 86% of eyes, after a mean of 2.3 surgeries per eye. Although visual acuity at presentation was very poor (hand movement or worse), in 50%, a final visual acuity of 20/200 or better was achieved. Analyzing the surgical details of these cases, they recommend performing lensectomy, a complete vitrectomy, and silicone oil tamponade in these difficult cases. Finally, A. Bor'i et al. have studied the surgical treatment of traumatic macular holes and report equal rates of anatomical closure and visual improvement with perflouropropane gas and silicone oil tamponade.

Another common theme in this special issue is the innovative use of drugs and technologies for retinal detachment surgery. X. Chen et al. investigated the combination of ketorolac with local anesthesia in patients undergoing retinal detachment surgery. They found that its addition resulted in reduced postoperative pain and the need of additional analgesia, as well as nausea and vomiting. A. N. Kulikov et al. also compared postoperative 360-degree laser retinopexy using a navigated pattern laser system, single-spot slit-lamp laser delivery, and single-spot indirect ophthalmoscope laser delivery. They have shown that the navigated pattern approach allows for more rapid treatment time and reduced pain in performing this procedure.

A basic science study by Z. Dvashi et al. investigated the use of OM-101, an inhibitor of TAK1, as a potential therapy for reducing the fibrotic response of proliferative vitreoretinopathy in an animal model. In this study, OM-101 was found to be safe and effective in reducing the incidence of retinal detachment, the number of proliferating and migrating retinal pigment epithelial cells, and the degree of fibrotic response. These results indicate that OM-101 may have the potential to improve or prevent proliferative vitreoretinopathy in patients with retinal detachment and could possibly be developed into a therapeutic modality in the future.

This special issue also includes several review articles. S. Mohamed et al. provide a thorough overview of the evolution of pars plana vitrectomy from 20-gauge instrumentation to the development of 27-gauge instrumentation, along with a discussion of the advantages and disadvantages of small-gauge vitrectomy, available instrumentation, and techniques. C. Alovisi et al. detail the development of vitreous substitutes, discussing both currently available types and those under development that may change the way retinal detachment surgeries are performed in the future. Finally, A. Nemet et al. reviewed recent innovations in surgical management of rhegmatogenous retinal detachment and proliferative vitreoretinopathy, focusing on the most recent literature, and discussed scleral buckling, vitrectomy, and pneumatic retinopexy techniques.

The guest editors would like to thank the authors of all the papers submitted to this special issue. The editors also wish to thank the many reviewers, who devoted their time, energy, and expertise and whose insightful comments helped improve the manuscripts selected for this special issue. We hope that the readers of this special issue will enjoy reading it and find its contents interesting and clinically valuable.

*Elad Moisseiev* *Anat Loewenstein* *Ala Moshiri* *Glenn Yiu*

